# Mask-Related Motion Artifact on 99mTc-MIBI SPECT: Unexpected Pitfalls of SARS-CoV-2 Countermeasures

**DOI:** 10.3390/diagnostics11081426

**Published:** 2021-08-06

**Authors:** Paweł Cichocki, Zbigniew Adamczewski, Jacek Kuśmierek, Anna Płachcińska

**Affiliations:** 1 Department of Nuclear Medicine, Medical University of Lodz, 92-216 Lodz, Poland; pawel.cichocki@office365.umed.pl (P.C.); jacek.kusmierek@umed.lodz.pl (J.K.); 2 Department of Quality Control and Radiation Protection, Medical University of Lodz, 92-216 Lodz, Poland; anna.plachcinska@umed.lodz.pl

**Keywords:** artifacts, nuclear medicine, myocardial perfusion imaging, technetium Tc 99m Sestamibi, SARS-CoV-2

## Abstract

A 61-year-old man was referred for myocardial perfusion scintigraphy (MPS) by an occupational physician to exclude coronary artery disease (CAD). The patient had a complete left bundle branch block (LBBB) that rendered the routine exercise stress test non-diagnostic, but otherwise had no history of heart diseases, good stress tolerance with no symptoms of angina, and no abnormalities in transthoracic echocardiogram, apart from contraction patterns typical for LBBB. Initial MPS, performed using technetium-labeled Sestamibi on a Discovery NM 530c camera equipped with solid-state semiconductor detectors, revealed a significant stress-induced ischemia that did not match the good overall condition of the patient. A motion detection procedure revealed significant heart motion in Z-axis during the stress study. Upon inquiry, the patient reported breathing difficulties caused by the mandatory mask, which slipped into an uncomfortable position during the study. Repeated acquisition, without motion artifacts, revealed no features of ischemia.

**Figure 1:**
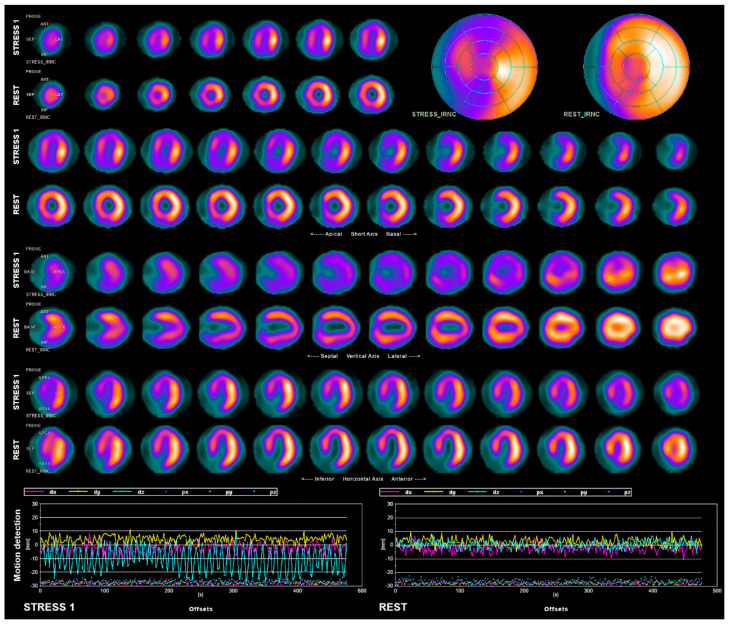
61-year-old male patient, a sailor, was referred for myocardial perfusion scintigraphy (MPS) by an occupational physician, in order to exclude coronary artery disease (CAD). Patient had a history of complete left bundle branch block (LBBB) that rendered a treadmill stress test (used as routine screening for job qualification) non-diagnostic. Apart from LBBB, patient had no history of heart diseases, diabetes, or uncontrolled hypertension and had good stress tolerance, with no symptoms of angina. Transthoracic echocardiogram revealed no abnormalities, apart from contraction patterns typical for LBBB. MPS was performed on GE Healthcare camera Discovery NM 530c, equipped with solid state cadmium-zinc-telluride (CZT) detectors, in a two-day rest/stress protocol, using 3 MBq/kg (0.08 mCi/kg) technetium-labeled Sestamibi (99mTc-MIBI) administered intravenously on each day. Image acquisition was performed in prone position for 8 min, 60 min after radiopharmaceutical administration. Stress test was carried out according to Bruce protocol. Patient completed stage 3 with a workload of 10.7 METs. The test was stopped after completing the protocol and reaching the target heart rate of 136 bpm, which was 85% of an age-predicted maximum rate. Blood pressure incremented normally in response to stress. The patient reported no symptoms during the test. The electrocardiogram (ECG) was non-diagnostic due to LBBB. Initial MPS images at rest revealed a mild perfusion defect in the area of an apex and inferoseptal wall of the left ventricle (LV). Stress images, however, revealed a significant stress-induced ischemia in the area of an anterior wall, septum, and inferior wall of LV. No issues were found during routine quality control of the study, which consisted of evaluation of sub-diaphragmal radiopharmaceutical activity and patient positioning on the camera. Due to the fact that stress images seemed distorted and findings did not match the good condition of the patient, quality control was expanded by motion detection, even though no patient motion was observed by the technician. Listmode data were reframed into 5 s frames and evaluated using motion detection and correction software for Alcyone (Xeleris 4.0). Evaluation revealed significant heart motion in the Z-axis throughout the entire stress study (up to 30 mm). Upon inquiry, patient reported that his mask slipped into an uncomfortable position during the study and caused difficulties with breathing. Motion artifact observed in the study was most likely caused by deeper breathing due to mask-induced discomfort and dyspnea. Motion artifacts were considered too significant for software motion correction; therefore, the stress acquisition was repeated.

**Figure 2:**
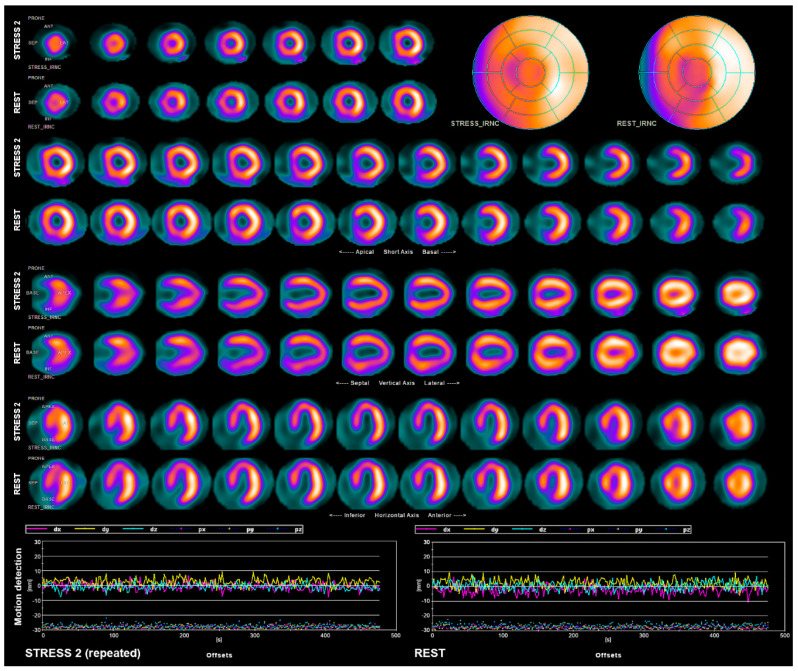
Stress acquisition was repeated with fixed mask position (~20 min after the first study), without any notable artifacts. Evaluation of the repeated study revealed no features of stress-induced ischemia. The only finding was a mild, fixed perfusion defect in an area of an apex and inferoseptal wall of LV, more pronounced in the rest study, which is common in LBBB [1,2]. Discovery NM 530c camera is susceptible to motion-related artifacts [3]. In normal circumstances, short acquisition time minimizes their likelihood and impact [4,5,6]. However, in times of the pandemic, mandatory masks can cause discomfort and breathing difficulties, which can increase the incidence and severity of motion artifacts. Motion detection on this camera is more difficult and time-consuming compared to traditional cameras [7] and requires additional data processing. For this reason, it is not performed routinely for every study. However, it is advised to perform it at least whenever a significant difference between perfusion in stress and rest studies is observed, especially when study result does not agree with the patient’s condition. Nuclear medicine technologist plays a major role in proper acquisition of the study and should ensure comfortable positioning of the patient, including the position of the face mask, to avoid such artifacts. Technologist should also closely monitor the patient and inform the physician if any patient motion occurred during the study, as motion detection procedure is not performed routinely, unlike in case of traditional SPECT gamma cameras. As shown in this case, mask-related motion artifacts can generate a false-positive MPS result that would qualify the patient for an unnecessary invasive study: coronary angiography.

## Data Availability

Data and material are available on reasonable request.
